# Distance-decay functions of travel to work trips in India

**DOI:** 10.1016/j.dib.2018.09.096

**Published:** 2018-10-02

**Authors:** Rahul Goel

**Affiliations:** UKCRC Centre for Diet and Activity Research (CEDAR), MRC Epidemiology Unit, University of Cambridge School of Clinical Medicine, Cambridge, UK

## Abstract

In 2011, for the first time, Census in India reported travel distance and mode of travel for the workers. The distance reported is in the form of aggregate counts for each mode of travel in 7 distance bins (0–1 km, 2–5 km, 6–10 km, 11–20 km, 21–30 km, 31–50 km, and >50 km). In this data article, methods are described to model categorical count data as distance-decay functions using continuous probability distributions. The distributions have been developed for 8 categories of modes—walk, cycle, motorised two-wheelers, car, tempo/auto rickshaw/taxi, bus, train, and all modes combined, for the 33 mainland states of India and all states combined. Distance for walk is modelled using exponential distribution, and for all the other modes using lognormal or Weibull distribution. For estimating parameters of the distributions, chi-square minimization has been used in a spreadsheet program. The data presented includes parameters of the 272 (34 × 8) probability distributions as well as descriptive statistics of these distributions.

**Specifications table**

**Table:** 

Subject area	*Civil Engineering*
More specific subject area	*Transportation Planning*
Type of data	*Tables*
How data was acquired	*Census of India website*
Data format	*Table in document and*Supplementary csv files
Experimental factors	
Experimental features	*Fitting of parametric probability distributions on categorical count data*
Data source location	*33 states of India*
Data accessibility	*Data in the article and*Supplementary material

**Value of the data**

• The data presented in this article includes the parameters of distance decay functions for 8 categories of travel modes in the forms of exponential, lognormal or Weibull distributions
• The data also includes descriptive statistics of travel distance for each mode in the 33 mainland states of India
• This is the first such travel-related data available for the whole of India
• The data will find use in multiple transport-related research as well as policy making, and the method described is generic and can be applied to estimate distance-decay functions at district level

## 1 Data

The commute distance data reported by the census is in the form of aggregate counts of workers classified into 7 distance bins for each mode. The count data will be modelled as continuous probability distribution functions to estimate mean distance travelled by each mode in each state. The data presented has been used for developing an accident prediction model for the states of India [5].

## 2 Materials and methods

In 2011, India had 28 states and 7 Union Territories (UTs). While the former has their own elected governments at the state levels, the latter are governed directly by federal government, and are usually much smaller in size than the states. The average population of the UTs is 2.9 million while that of the states is 41 million. Two of the UTs are islands, Andaman and Nicobar Island in the east and Lakshadweep in the west, and contribute 0.04% of the total population of the country. These were excluded from this analysis. The remaining 28 states and 5 UTs will be referred to as 33 states henceforth. In addition, the analysis includes all states combined, referred to as India.

Census in independent India has been conducted every decade from 1951 using personal interviews and covers the whole population. In 2011, Census introduced two questions regarding the commute of workers [1]. The two questions on commuting included mode of travel and one-way distance (in kilometres) from residence to place of work. There are 9 options for the travel modes: (1) walk, (2) cycle, (3) moped/scooter/motorcycle, (4) car, (5) tempo/auto rickshaw/taxi, (6) bus, (7) train, (8) water transport, and (9) any other, and an option of ‘No travel’. Category 3 is referred to as motorised two-wheelers (2W), and category 5 as para-transit modes or Intermediate Public Transport (IPT) such as three-wheeled auto rickshaws, common across India (for their description see [6,9]).

These questions were asked from a subset of all the workers—the category called ‘other workers’. These are defined as the workers other than those involved in economic activities such as cultivation, agriculture labour, or a household–based industry. Within urban areas, the workers are classified as working in a household-based industry if the business is conducted by the household members within the premises of their household. In rural areas, workers are classified as household-based if the industry is conducted within the village. If the person was engaged in more than one economic activity during the last year, this question was asked with reference to the main economic activity. The category of ‘other workers’ represent 42% of all the workers in India [2].

Among the 9 options of travel modes, only one could be selected by a worker. The question on mode thus disregards the multimodal characteristics of some of the trips, and census provides no details in this regard [3]. Thus, the working assumption is that the respondents informed their main mode of travel—the one using which they covered the longest travel distance. Since the census is conducted using personal interviews, it is possible that these questions, in some cases, were answered by proxy respondents, for instance, by other members of the household. However, no such information is available from Census to account for this bias.

For each mode, census has reported mode-specific count of workers classified into 7 distance categories: 0–1 km, 2–5 km, 6–10 km, 11–20 km, 21–30 km, 31–50 km, and >50 km. For walk, counts have been reported for 3 categories up to 10 km, and for cycle, 5 categories up to 30 km [1]. Table 1 presents this data for all India. The data has been reported only at the aggregate level of states and districts, with a further classification into rural, urban and total. In this article, total data (urban plus rural) has been used at the state level. All modes combined is also included as an additional category. ‘Water transport’ and ‘any other’ categories were excluded. These two categories were reported by 1.2% of those travelling by one of the 9 travel modes. In total, this article presents analysis of 8 travel mode categories in 33 states plus all India.

**Table 1:** Counts of workers classified by mode of travel and distance.

**Mode**	**0–1 km**	**2–5 km**	**6–10 km**	**11–20 km**	**20–30 km**	**31–50 km**	**> 50 km**
**Walk**	23,745,884	14,297,484	7,223,200	–	–	–	–
**Bicycle**	3,707,522	13,030,315	5,324,105	1,944,975	2,265,692	–	–
**2W**	3,181,808	9,692,650	5,695,234	3,443,182	1,115,748	765,674	332,926
**IPT**	443,486	2,106,958	1,536,981	892,688	308,772	243,986	155,089
**Bus**	735,997	4,303,131	5,399,703	4,745,456	2,381,647	2,193,470	2,327,868
**Car**	451,265	1,416,759	11,73,018	1,015,482	472,356	391,698	274,099
**Train**	242,561	490,647	692,189	1,059,169	857,873	1,147,380	1,929,085
**All Modes**^a^	32,699,104	45,752,670	27,365,589	13,323,946	7,510,524	4,864,535	5,237,375

a All modes consist of the 7 travel mode categories in the table as well as water transport and ‘any other’.

To estimate mode-specific average distance, the count data is modelled as continuous probability distributions. Such distributions, for distance, are often referred to as distance-decay functions [7], [8], [12]. The decay function for walking is often modelled with an exponential distribution. This implies that the likelihood is the highest for walking trips with distance close to zero and this likelihood reduces thereafter. In case of all the other modes, the peak, however, reaches at a point away from zero, followed by a long tail towards longer distance. Such variations in probabilities are often expressed using lognormal or Weibull distributions.

In their original form, exponential, lognormal as well as Weibull have domains reaching up to infinity. This means that an integral of these distributions from zero to infinity is unity. Since commute travel distance has a finite maximum value, for each of the distributions, their truncated forms were used. Without the truncations, distributions are likely to overestimate the average distance. Mathematically, truncation implies that probability density functions integrate to unity within a restricted domain i.e. a finite maximum value.

The truncated forms of cumulative distribution functions are shown in the Eqs. (1), (2), (3). Each of the distribution is expressed using two parameters, α and β, and the combination of two parameters is specific to a given combination of distribution type (denoted in the subscript by l: lognormal; w: Weibull; e: exponential), state (denoted by s), and mode of travel (denoted by m). In case of exponential function, the subscript for mode has not been used as this distribution is applicable only for walking.

Lognormal distribution:

(1) $$F\left(\mathrm{x|}\alpha_{l,m,s},\beta_{l,m,s}\right)=\left(\frac{1}{2}+\frac{1}{2}\mathrm{erf}\left[\frac{\ln x-\alpha_{l,m,s}}{\sqrt{2}\beta_{l,m,s}}\right]\right)/F\prime (D_{\max,m}|\alpha_{l,m,s},\beta_{l,m,s}),$$

where, erf is the Gauss error function

Weibull distribution:

(2) $$F\left(\mathrm{x|}\alpha_{w,m,s},\beta_{w,m,s}\right)=\left(1-e^{{-\left(\frac{x}{\beta_{w,m,s}}\right)}^{\alpha_{w,m,s}}}\right)/F\prime (D_{\max,m}|\alpha_{w,m,s},\beta_{w,m,s})$$

Exponential distribution:

(3) $$F\left(\mathrm{x|}\alpha_{e,s},\beta_{e,s}\right)=(1-\beta_{e,s}e^{-\left(\alpha_{e,s}x\right)})/F\prime (D_{\max}|\alpha_{e,s},\beta_{e,s})$$

In the above equations, $F\prime \left(D_{\max}\right)$ is the normalising factor which ensures that the integration of the distribution from 0 to $D_{\max}$ equals unity. It is calculated as the cumulative probability of the untruncated distribution at $x=D_{\max,m}$, where the subscript m denotes that this distance is specific to a mode. The objective is to find the parameters *α* and β.

For exponential distribution, mean and standard deviation were analytically derived. For lognormal and Weibull distributions, I used analytical forms of mean and standard deviation values reported in the literature ([4] for Weibull and [11] for lognormal). Crénin [4] reported an R script for estimating mean and standard deviation, given the parameters α, β and $D_{\max,m}$ (**see** Box 1 for expressions of mean and standard deviations for the three distributions).

Box 1 **Mean and standard deviation calculations for the three distributions.**

1) **Lognormal distribution truncated at distance D** [11]
  Cumulative Distribution function: $F\left(\mathrm{x|}\alpha ,\beta_{l,m,s}\right)=\left(\frac{1}{2}+\frac{1}{2}\mathrm{erf}\left[\frac{\ln x-\alpha }{\sqrt{2}\beta }\right]\right)/F(\mathrm{D|}\alpha ,\beta )$, where, erf is the Gauss error function Mean (μ): $e^{(\alpha +\frac{1}{2}\beta^{2})}\Phi (A-\beta )/F(\mathrm{D|}\alpha ,\beta )$ Standard Deviation (σ): $\sqrt{e^{(2\alpha +2\beta^{2})}\Phi (A-2\beta )/F(\mathrm{D|}\alpha ,\beta )}$
  where A=(lnD−α)/β and Φ is the cumulative distribution function of the standard normal distribution (i.e. *N* (0,1)).
2) **Weibull Distribution:** See right-truncated two-parameter Weibull distribution in Crénin [4]. Mean and standard deviation calculated using R script reported in the study.
3) **Exponential distribution:** $F\left(\mathrm{x|}\alpha ,\beta \right)=(1-\beta e^{-\left(\alpha x\right)})/F(D|\alpha ,\beta )$

Mean (μ): $\frac{\left(1-\left(D\alpha +1\right)e^{-D\alpha }\right)\beta }{\alpha F(D|\alpha ,\beta )}$

Standard deviation (σ): $\frac{\beta }{\alpha^{2}F(D|\alpha ,\beta )}{(e}^{-D\alpha }\left(-\alpha \left(\mu -D\right)\left(\alpha \left(\mu -D\right)-2\right)-2\right)+\alpha \mu \left(\alpha \mu -2\right)+2)$

The counts in the 7 distance bins specific to each mode are referred to as $n_{\mathrm{obs},m,s}^{1}$, $n_{\mathrm{obs},m,s}^{5}$, $n_{\mathrm{obs},m,s}^{10}$, $n_{\mathrm{obs},m,s}^{20}$, $n_{\mathrm{obs},m,s}^{30}$, $n_{\mathrm{obs},m,s}^{50}$ and $n_{\mathrm{obs},m,s}^{50+}$, and the total number of workers corresponding to each mode as $n_{\mathrm{obs},m,s}^{\mathrm{total}}$, where subscript *obs* refers to the observed numbers, *m* refers to the mode of travel, *s* refers to the state, and the numbers in superscript refer to the distance bin. The number of workers within each distance bin modelled by distribution function are referred to as $n_{\mathrm{mod},m,s}^{1}$, $n_{\mathrm{mod},m,s}^{5}$, $n_{\mathrm{mod},m,s}^{10}$, $n_{\mathrm{mod},m,s}^{20}$, $n_{\mathrm{mod},m,s}^{30}$, $n_{\mathrm{mod},m,s}^{50}$ and $n_{\mathrm{mod},m,s}^{50+}$, thus replacing ‘*obs*’ by ‘*mod*’ in the subscripts.

To estimate the parameters α and β, an optimisation problem is setup, where the objective is to minimise the chi-square statistics given in Eq. (4).

(4) $$\chi^{2}=\sum_{i}\frac{({n_{\mathrm{obs},m,s}^{i}-n_{\mathrm{mod},m,s}^{i})}^{2}}{n_{\mathrm{mod},m,s}^{i}}$$

where, index i refers to 1, 5, 10, 20, 30, 50, and 50+, and $n_{\mathrm{mod}}^{1}$ = $n_{\mathrm{obs}}^{\mathrm{total}}\times F\left(x=1\;\mathrm{km}\right)$, $n_{\mathrm{mod}}^{5}$ = $n_{\mathrm{obs}}^{\mathrm{total}}\times (F\left(x=5\;\mathrm{km}\right)-F\left(x=1\;\mathrm{km}\right))$, and so on. Here, F(x=1km) refers to the probability of modelled counts less than or equal to 1 km and (F(x=5km)−F(x=1km)), refers to the percentage of counts greater than 1 km but less than 5 km. F(x) refers to the cumulative probability distribution presented in Eqs. (1), (2), (3). The optimisation problem is to find the combination of parameter values α and β for which the value of $\chi^{2}$ statistic is minimised.

To obtain the solutions, I used the Solver tool of Microsoft Excel spreadsheet program. In Solver, GRG (generalised reduced gradient: [10]) non-linear algorithm was used for optimisation. The solution of algorithm is sensitive to the selection of starting points for α and β. From a preliminary analysis for all the modes, it was clear that for a given mode and a given distribution, the values of these parameters belonged to a narrow range. Therefore, any outlying solution in terms of parameters would be easy to detect. The value of 1 was used as starting point for the both the parameters as a positive value.

In census data, $D_{\max}$ is 10 km for walk and 30 km for cycle. For all the other modes (2W, car, IPT, bus, and train), the last bin is for distance greater than 50 km and is open ended (>50 km). I assumed 100 km as $D_{\max}$ for 2 W, 200 km for car and train, 100 km for IPT, and 100 km for bus. To test the sensitivity of these assumptions, I estimated mean distance assuming $D_{\max}$ of 150 km and 300 km in case of car. The maximum difference in means is 0.5 km in the two assumptions.

**Fig. 1:**
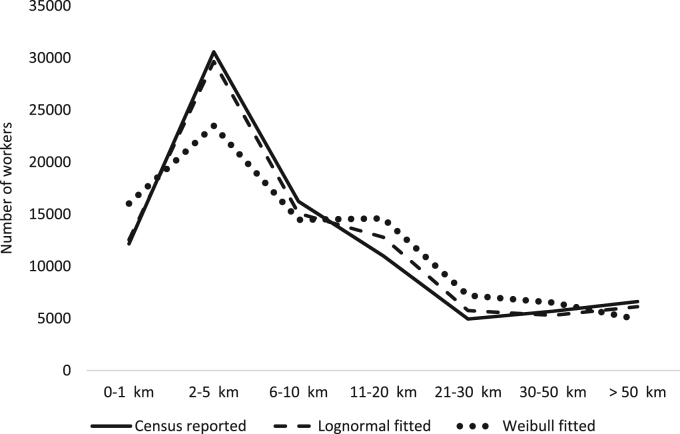
Comparison of lognormal and Weibull distribution fit for Cars in the state of Assam.

During the initial analysis, it was found that, for walking, exponential distribution provides the perfect fit for all the states, indicated by minimum $\chi^{2}$statistic value of zero. Thus, for walk, the number of modelled counts were equal to their corresponding observed values in the three distance bins. For all the other modes, except cycle, either lognormal or Weibull distributions provided a good fit. First, lognormal model was fitted for all the modes in all the states. Next, those with a poor fit were fitted with Weibull distribution. To identify a poor fit, I used Pearson correlation between the modelled and observed number of counts in the bins. It is because $\chi^{2}$statistic is not a normalised value and hence does not facilitate comparison across different cases. The distinction between good and poor fit was easy to identify as good fit provided a correlation of 0.99 or more while the poor fit was much lesser. Fig. 1 presents an example of fitting two different distributions for Cars in the state of Assam. It can been that lognormal is a much better fit than Weibull. The former has a $\chi^{2}$ value of 560 while the latter has a value of 5574, thus lognormal is clearly a better distribution fit in this case.

**Fig. 2:**
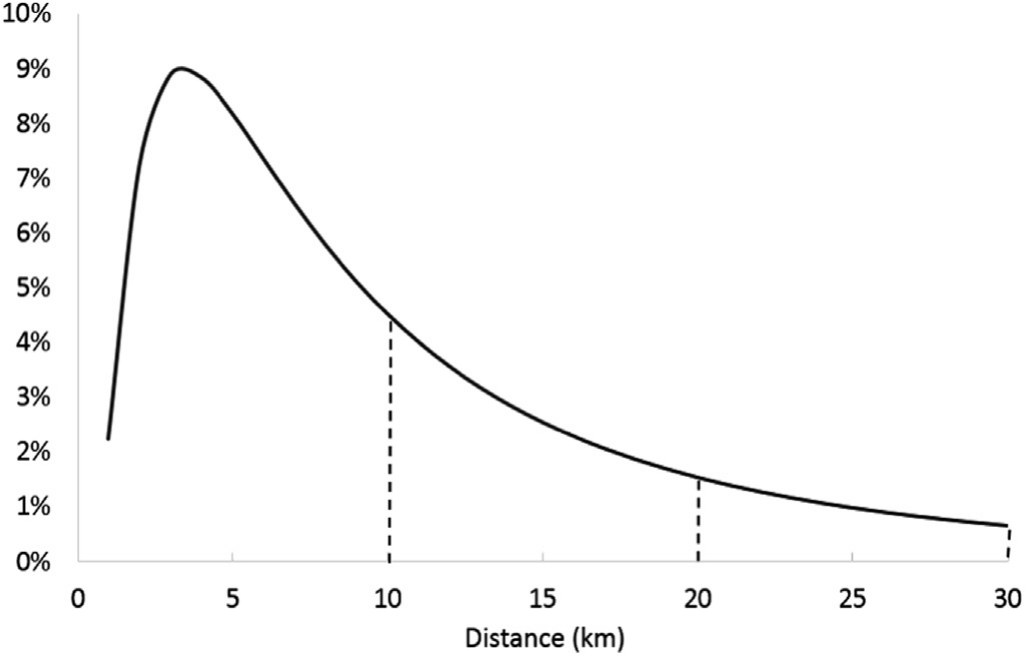
Hypothetical lognormal distribution for cycling.

In case of cycle, it was observed that in most states the counts in 21–30 km bin are more than, or in some cases almost the same as, the preceding bin of 11–20 km. Note that both bins are of equal size (10 km). There are some exceptions, such as Delhi, where number of cycle trips in the last bin (21–30 km) are 30% of those in the preceding bin (11–20). Any distribution for cycle distance is likely to have a negative slope at this distance range (>10 km) (see, for example, Fig. 2. Therefore, with the two bins of equal size (10 km), it is not possible to have more number of trips in 21–30 km range than 11–20 km. There is clearly some discrepancy in the data.

**Fig. 3:**
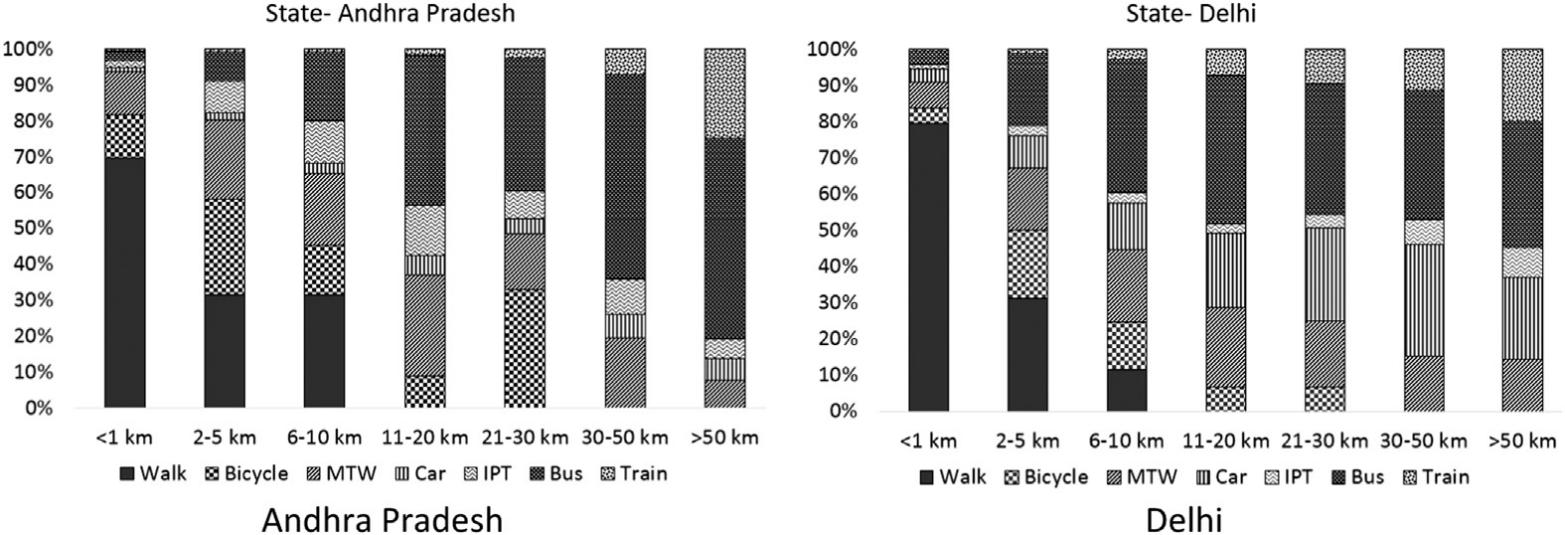
Relative shares of modes for the distance bins (notice a sudden increase in the share of cycling in 21–30 km bin in Andhra Pradesh compared to Delhi).

An alternative way to highlight this discrepancy is by observing the relative shares of different modes within each distance bin. For bins of longer trip distance, the relative share of motorised modes is expected to increase, and that of non-motorised modes expected to decrease. Fig. 3 presents this data for one of the states (Andhra Pradesh) where 21–30 km bin has higher number of counts than 11–20 km, and for Delhi, where the reverse is true. For 21–30 km, the relative share of cycle trips (chequered pattern) in Andhra Pradesh increases abruptly, while this transition is gradual in case of Delhi.

To correct this discrepancy, the two bins (10–20 km and 20–30 km) were combined into one large bin of 10–30 km. Thus, for cycling, the revised observed data consists of 4 bins— $n_{\mathrm{obs}}^{1}$, $n_{\mathrm{obs}}^{5}$, $n_{\mathrm{obs}}^{10}$, and $n_{\mathrm{obs}}^{30\prime }$, in which the last bin refers to number of trips from 10–30 km. After this modification, the distribution fit increased considerably. Using the fitted distributions, the number of trips were estimated for the two bins— 10–20 km and 20–30 km. It was observed that a large number of trips in 20–30 km bin are shifted to 10–20 km. In case of Delhi, the distribution was fitted without any modification in the bins, and a perfect fit was obtained.

**Fig. 4:**
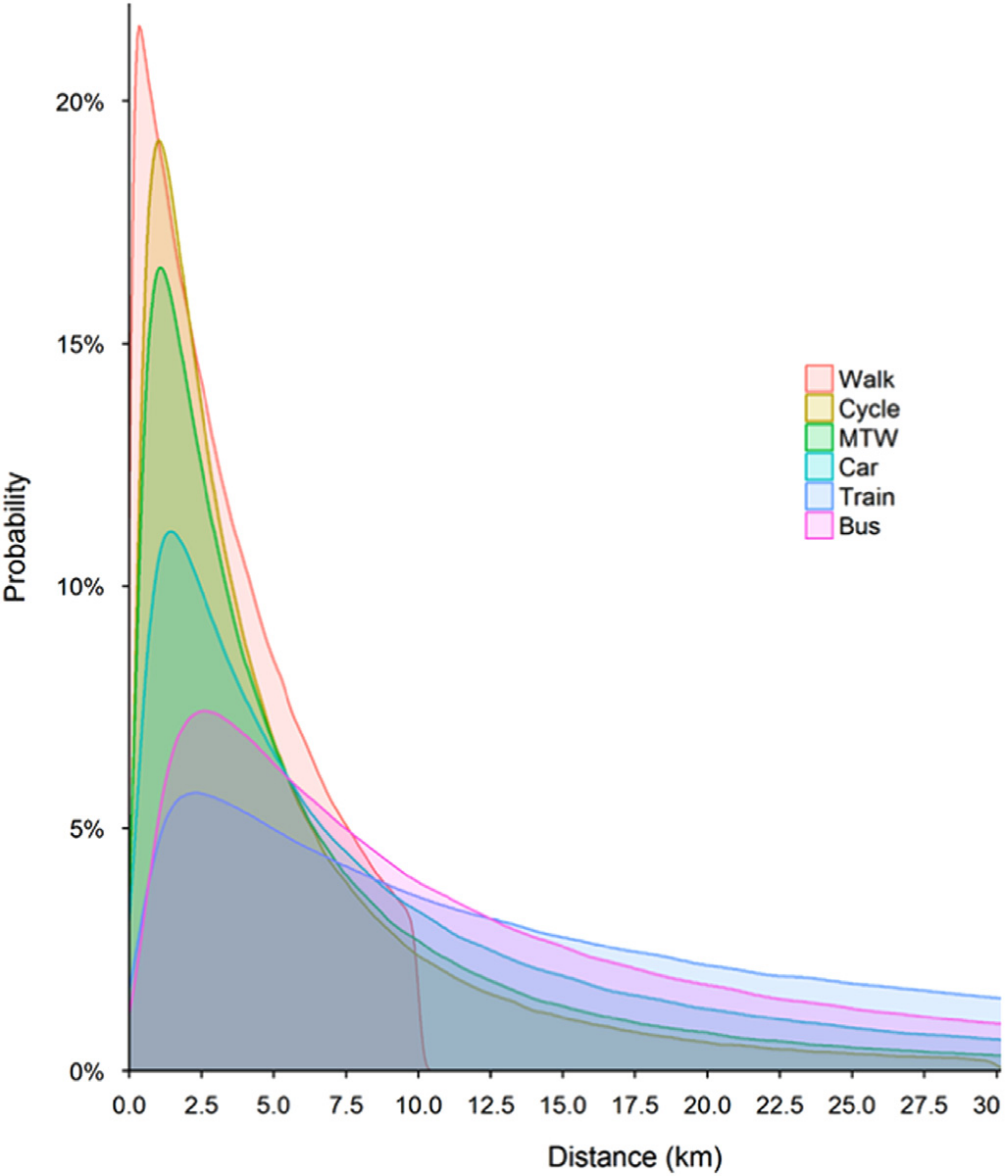
Distance-decay functions for all India.

Once the parameters of each mode in each state are known, I calculated average distance as well as the total distance travelled by the mode by all workers. Table 2 presents average and standard deviation of the distance. Fig. 4 presents distance-decay functions of six modes for all India. The maximum distance on x-axis is 30 km for a better representation. The distribution parameters and detailed descriptive statistics for each mode and for each state are presented in the Supplementary material.

**Table 2:** Average (standard deviation) trip distance by mode in km for India and 33 states.

**State**	**All modes**	**Walk**	**Bicycle**	**Bus**	**Car**	**IPT**	**2W**	**Train**
India	10.1 (16.5)	2.1 (2.3)	5.4 (7.8)	21.1 (26)	15.6 (28.4)	10 (16.2)	8.2 (14.2)	51.9 (62)
Andhra Pradesh	10.5 (16.8)	2.4 (2.4)	5.1 (7.4)	23.4 (27.7)	17.0 (26.8)	10 (15.7)	8.6 (15.1)	77.7 (77.8)
Arunachal Pradesh	5.0 (12.2)	1.8 (2.4)	4.1 (6.7)	21.9 (26.3)	12.3 (27.1)	8.9 (15.4)	7.3 (14.7)	23.6 (46.4)
Assam	7.3 (13.5)	1.5 (2)	4.2 (6.4)	33.8 (33.5)	14.6 (29)	8.1 (13.5)	7.2 (13.1)	55.5 (79.7)
Bihar	9.3 (15.6)	2.4 (2.5)	6.0 (8.5)	23.9 (34.9)	16.4 (31.2)	8.9 (14.4)	9.3 (16.3)	55.1 (76.4)
Chandigarh	7.0 (11.5)	1.9 (2.1)	5.7 (7.6)	17.3 (22.8)	9.3 (15.8)	7.9 (12.3)	6.5 (9.6)	36.6 (50.4)
Chhattisgarh	7.1 (12.9)	1.5 (1.9)	5.2 (7.5)	30.1 (41.2)	17.3 (32.6)	10.5 (16.6)	8.2 (14.4)	38.1 (57)
Dadra & Nagar Haveli	4.5 (9.1)	0.9 (1.7)	3.5 (5)	12.7 (17.8)	9.5 (18)	6.7 (10.2)	6.4 (11.3)	61.4 (85.1)
Daman & Diu	5.1 (11)	1.0 (1.5)	2.7 (3.8)	32.7 (32.4)	7.3 (13.9)	6.7 (10.6)	5.6 (10)	55.1 (79.8)
Goa	10.0 (16.6)	2.0 (2.3)	5.2 (7.5)	16.2 (21.8)	13.4 (25.2)	11.6 (19.6)	10.2 (17.4)	30 (45.5)
Gujarat	7.7 (13.4)	1.9 (2.2)	4.4 (6.3)	24.2 (28.3)	14.6 (26.6)	8.5 (13.5)	6.7 (11.4)	41.3 (57.8)
Haryana	12.9 (19.2)	2 (2.3)	5.4 (7.6)	38.9 (36.7)	17.7 (31.3)	10.2 (15.7)	8.4 (14.6)	53.3 (69.7)
Himachal Pradesh	9.1 (17.8)	2.1 (2.2)	5.4 (7.6)	19.2 (24.5)	13.1 (26)	13.5 (22.4)	9.1 (15.8)	40.9 (64.4)
Jammu & Kashmir	12.5 (23.2)	2.6 (2.5)	6.3 (8.7)	22.4 (26.9)	16.3 (28.7)	12.8 (20.2)	8.8 (14.4)	66.2 (88.6)
Jharkhand	7.9 (13.8)	2.2 (2.2)	6.3 (8.6)	29.5 (41.9)	13.8 (26.1)	9.4 (14.3)	7.5 (12.8)	55.7 (76.3)
Karnataka	10.6 (16.9)	2.2 (2.3)	5.7 (8.1)	18.4 (23.8)	15.7 (27.9)	12.6 (20.3)	8.6 (14.8)	64.8 (70.1)
Kerala	8.7 (15.8)	1.7 (2)	4.3 (6.1)	12.4 (17.6)	11.1 (20.8)	6.4 (11.4)	7.7 (12.4)	81.4 (99.4)
Madhya Pradesh	7.5 (13.4)	2.2 (2.3)	5.5 (7.8)	26.4 (29.8)	15.3 (29.5)	9 (14.6)	7.5 (13.4)	76.6 (77)
Maharashtra	10.2 (18.5)	2.2 (2.3)	5.0 (7.2)	17.0 (22.6)	14.5 (22.1)	10.3 (17.2)	8.8 (15.2)	38.7 (55.2)
Manipur	8.3 (16.2)	2.1 (2.6)	5.3 (7.7)	25.8 (35.2)	13.5 (26.3)	8 (13.2)	6.8 (11.5)	23.5 (43.7)
Meghalaya	8.5 (15)	1.8 (2.2)	4.7 (7.1)	36.0 (34.2)	14.8 (28.8)	6.9 (11.9)	9.6 (17.5)	26.5 (50.9)
Mizoram	4.3 (9.7)	0.9 (1.8)	3.4 (5.5)	8.8 (14.8)	10.3 (23.8)	4.3 (8.5)	4.5 (8.8)	24.8 (49.9)
Nagaland	4.6 (10.2)	1.2 (2)	3.2 (4.9)	13.4 (19.4)	10.4 (22.4)	6.7 (12.1)	5.7 (10.7)	21.6 (44.7)
Delhi	8.8 (14.2)	1.6 (1.9)	5.9 (8)	12.0 (15.7)	14.0 (18.6)	13.5 (21.6)	10.9 (17.4)	19 (23.8)
Odisha	9.5 (15.8)	2.2 (2.3)	6.2 (8.9)	33.6 (46)	19.7 (36.3)	12 (19.3)	9.9 (17.4)	60 (82.5)
Puducherry	8.8 (14.6)	1.9 (2.2)	4.8 (6.9)	18.1 (23.6)	14.9 (28.2)	9.1 (15.9)	7.1 (12)	38.1 (59.5)
Punjab	8.8 (14.9)	2.3 (2.5)	5.8 (8.2)	30.8 (32.5)	13.3 (24.5)	9.1 (14.6)	7 (12.2)	47.4 (69.7)
Rajasthan	10.4 (16.8)	1.9 (2)	5.1 (7.1)	32.8 (33.4)	15.8 (28.7)	9.4 (14.7)	7.9 (13.4)	75.2 (90.8)
Sikkim	5.6 (12.3)	1.7 (2.1)	7.0 (10.1)	14.6 (20.4)	12.6 (24.2)	9.9 (16.9)	9.7 (16.4)	36.6 (61.6)
Tamil Nadu	11.7 (18)	1.9 (2.2)	4.5 (6.6)	20.4 (25.5)	16.5 (29.4)	14.2 (23)	8 (13.6)	51.4 (61.6)
Tripura	6.1 (11.7)	1.7 (2)	3.9 (5.6)	24.3 (33.3)	16.9 (24.5)	7.2 (11)	7 (12.1)	18.9 (34.4)
Uttar Pradesh	10.6 (17)	2.3 (2.5)	6.6 (9.3)	36.1 (34.8)	16.3 (25.9)	10.9 (17.1)	9.6 (16.4)	85.2 (81.8)
Uttarakhand	8.3 (14.4)	1.9 (2.2)	5.5 (7.8)	29.5 (31.6)	16.6 (30.5)	9.2 (14.1)	7.3 (12.8)	38.5 (60.5)
West Bengal	12.1 (18.5)	2 (2.4)	5.0 (7.5)	21.4 (26.2)	11.7 (21.8)	10.5 (17.6)	8.5 (15)	41.2 (54.7)

## 3 Conclusion

This article presents a method to fit continuous probability distributions (distance-decay functions) for the categorical count data reported by Census. Also presented is the descriptive statistics of travel distance by mode. This method can be further extended to estimate distance-decay functions at the smaller levels of jurisdictions such as districts (counties) or cities. Due to simplistic questions in the Census questionnaire about the mode of travel to work, it has been assumed that the Census respondents informed their main mode of travel—the one using which they covered the longest travel distance. Further, since the census in India is conducted using personal interviews, it is possible that these questions, in some cases, were answered by proxy respondents, for instance, by other members of the household. However, no such information is available from Census to account for this bias.
